# Triterpene Esters and Biological Activities from Edible Fruits of *Manilkara subsericea* (Mart.) Dubard, Sapotaceae

**DOI:** 10.1155/2013/280810

**Published:** 2012-12-20

**Authors:** Caio P. Fernandes, Arthur L. Corrêa, Jonathas F. R. Lobo, Otávio P. Caramel, Fernanda B. de Almeida, Elaine S. Castro, Kauê F. C. S. Souza, Patrícia Burth, Lidia M. F. Amorim, Marcelo G. Santos, José Luiz P. Ferreira, Deborah Q. Falcão, José C. T. Carvalho, Leandro Rocha

**Affiliations:** ^1^Laboratório de Tecnologia de Produtos Naturais, Faculdade de Farmácia, Universidade Federal Fluminense, Rua Doutor Mário Viana 523, Santa Rosa, 24241-000 Niterói, RJ, Brazil; ^2^Departamento de Tecnologia Farmacêutica, Faculdade de Farmácia, Universidade Federal Fluminense, Rua Doutor Mário Viana 523, Santa Rosa, 24241-000 Niterói, RJ, Brazil; ^3^Departamento de Biologia Celular e Molecular, Instituto de Biologia, Universidade Federal Fluminense, Outeiro São João Batista s/n, Centro 24020-141 Niterói, RJ, Brazil; ^4^Departamento de Ciências, Faculdade de Formação de Professores, Universidade do Estado do Rio de Janeiro, Dr. Francisco Portela 1470, P 24435-000 São Gonçalo, RJ, Brazil; ^5^Laboratório de Farmacognosia, Faculdade de Farmácia, Universidade Federal Fluminense, Rua Doutor Mário Viana 523, Santa Rosa, 24241-000 Niterói, RJ, Brazil; ^6^Laboratório de Pesquisa em Fármacos, Colegiado de Ciências Farmacêuticas, Universidade Federal do Amapá, Campus Universitário-Marco Zero do Equador, Rod. Juscelino Kubitschek de Oliveira, KM-02, Bairro Zerão, 68902-280 Macapá, AP, Brazil

## Abstract

*Manilkara subsericea* (Mart.) Dubard (Sapotaceae) is popularly known in Brazil as “guracica.” Studies with *Manilkara* spp indicated the presence of triterpenes, saponins, and flavonoids. Several activities have been attributed to *Manilkara* spp such as antimicrobial, antiparasitic and antitumoral, which indicates the great biological potential of this genus. In all, 87.19% of the hexanic extract from fruits relative composition were evaluated, in which 72.81% were beta- and alpha-amyrin esters, suggesting that they may be chemical markers for *M. subsericea*. Hexadecanoic acid, hexadecanoic acid ethyl ester, (E)-9-octadecenoic acid ethyl ester, and octadecanoic acid ethyl ester were also identified. Ethanolic crude extracts from leaves, stems, and hexanic extract from fruits exhibited antimicrobial activity against *Staphylococcus aureus* ATCC25923. These extracts had high IC_50_ values against Vero cells, demonstrating weak cytotoxicity. This is the first time, to our knowledge, that beta- and alpha-amyrin caproates and caprylates are described for *Manilkara subsericea*.

## 1. Introduction

**Figure 1:**
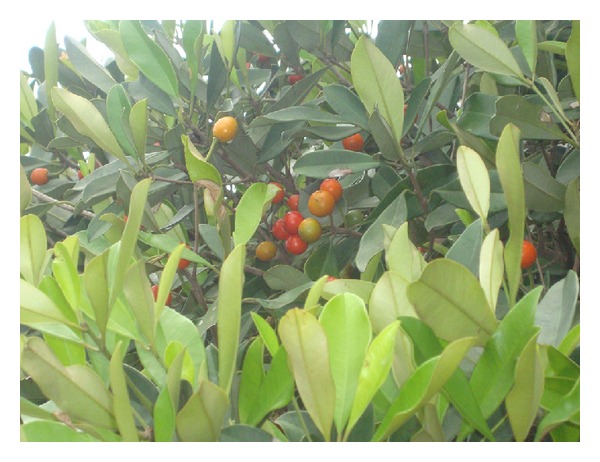
*Manilkara subsericea *(Mart.) Dubard, Sapotaceae at Restinga de Jurubatiba National Park (RJ, Brazil).

*Manilkara subsericea* (Mart.) Dubard (Sapotaceae) is popularly known in Brazil as “guracica,” “maçaranduba-pequena,” “maçaranduba-vermelha,” “maçarandubinha,” or “paraju” (Figure 1). This species is widely spread on the sandbanks of eastern Brazil, from the states of Espírito Santo to Santa Catarina. *M. subsericea* has edible fruits, being consumed *in natura*, and local population also use its wood for construction [1, 2]. Studies with species from the genus *Manilkara* indicated the presence of triterpenes [3], saponins [4], and flavonoids [5]. Several activities have been attributed to *Manilkara *spp., such as antimicrobial [6, 7], antiparasitic [8, 9], and antitumoral [10], which indicates the great biological potential of the genus.

On the present study, we evaluated the antibacterial and cytotoxicity activity of extracts from *Manilkara subsericea*. We also made the phytochemical characterization of the hexanic extract from edible fruits of *M. subsericea*.

## 2. Materials and Methods

### 2.1. Plant Material

Aerial parts with fruits of *Manilkara subsericea *(Mart.) Dubard (Sapotaceae) were collected at Restinga de Jurubatiba National Park (RJ, Brazil) in January 2009 and were identified by the botanist Dr. Marcelo Guerra Santos. A voucher specimen of *M. subsericea *was deposited at the herbarium of the Faculdade de Formação de Professores (Universidade do Estado do Rio de Janeiro, Brazil) under the register number RFFP 13.416.

### 2.2. Preparation of Extracts

Extracts were obtained from fruits, leaves, and stems. The *M. subsericea* freshly fruits (1.14 kg) were crushed and macerated with ethanol (EtOH) 96% (v/v) at room temperature until exhaustion. This ethanolic extract was concentrated in vacuum using a rotary evaporator to obtain ethanolic crude extract from fruits (170 g). This extract was dissolved into 500 mL EtOH/H_2_O 90% (v/v) mixture and partitioned with hexane (2 × 600 mL) to obtain, after evaporation of the hexanic portion, 14.0 g of hexanic extract from fruits (FH). The hydroalcoholic portion from this partition was evaporated in vacuum and resuspended in 500 mL distilled water. This aqueous suspension was successively partitioned with ethyl acetate (2 × 600 mL) and butanol (2 × 600 mL), furnishing, after evaporation, 4.5 g of ethyl acetate extract (FEA), and 6.8 g of butanol extract (FB) from fruits. Leaves (1.93 kg) and stems (0.96 kg) were individually dried at 40°C for two days, triturated and macerated with ethanol (EtOH) 96% (v/v) at room temperature until exhaustion. Each ethanolic extract was concentrated in vacuum using a rotary evaporator to obtain 530 g of ethanolic crude extract from leaves (LET) and 169.3 g of ethanolic crude extract from stems (SET). 

### 2.3. Analysis of FH by Gas Chromatography-Mass Spectrometry

The hexanic extractfrom fruits (FH) was analyzed by a GCMS-QP5000 (SHIMADZU) gas chromatograph equipped with a mass spectrometer using electron ionization, according to these experimental conditions: injector temperature, 270°C; detector temperature, 290°C; carrier gas, Helium; flow rate 1 mL/min; split injection with split ratio 1 : 50. The oven temperature was programmed from 60°C (isothermal for 3 min), with an increase of 10°C/min to 290°C, ending with a 59 min isothermal at 290°C. One microliter of the sample, dissolved in CHCl_3_ (1 : 100 mg/*μ*L), was injected into a ZB-5MS column (i.d. = 0.25 mm, length 30 m, film thickness = 0.25 mm). Mass spectrometry (MS) conditions were ionization voltage, 70 eV and scan rate, 1 scan/s. The identification was performed by comparison of the MS fragmentation pattern of the substances of FH with NIST mass spectra libraries. Quantitative analysis of the chemical constituents was performed by flame ionization gas chromatography (CG/FID), under same conditions of GC/MS analysis and percentages obtained by FID peak area normalization method.

### 2.4. Antimicrobial Activity 

#### 2.4.1. Microbial Strain

*Staphylococcus aureus *ATCC25923 and *Escherichia coli *ATCC36298, obtained from the culture collections of the Laboratório de Controle Microbiológico, Faculdade de Farmácia, Universidade Federal Fluminense, were used for the antibacterial activity experiments. Overnight cultures were prepared by inoculating approximately 2 mL Tryptic soy broth (TSB; Difco) with 2-3 colonies of each organism. Bacterial strains were cultured overnight at 37°C. Inocula were prepared by diluting overnight cultures in saline to approximately 10^8^ CFU/mL.

#### 2.4.2. Diffusion Disk Assay

Qualitative antimicrobial tests were carried out by disk diffusion method [11]. Briefly, a suspension of microorganism (10^8^ UFC/mL) was spread on the solid media plates of Muller-Hinton agar (Difco). Disks (6 mm in diameter) were impregnated, until saturation, with the ethanolic crude extracts from leaves and stems, hexanic, ethyl acetate, and butanol extracts from fruits. Then, disks were placed on the inoculated agar. Vancomycin (30 *μ*g) and ampicillin (30 *μ*g) were used as positive reference standards of the test. Disks impregnated with solvents used for solubilization of extracts were used as negative control. The inoculated plates were incubated at 37°C for 24 h. Antimicrobial activity was evaluated by measuring the zone of inhibition against the test organisms. Each experiment made in triplicate.

#### 2.4.3. Minimum Inhibitory Concentration (MIC)

A micro-dilution technique using 96 well micro-plates, as described by Eloff [12] was used to obtain MIC values of extracts against *S. aureus*. The method comprised of filling all the wells of a 96 well microplate with 100 *μ*L of Muller-Hinton broth (Vetec). Triplicates (100 *μ*L) of the samples (ethanolic crude extracts from leaves and stems, hexanic, ethyl acetate, and butanol extracts from fruits) at starting concentrations of 2 mg/mL in DMSO were introduced into the first well. Serial doubling dilutions were then performed, rejecting 100 *μ*L from each well and adding a mixture of test micro-organism (100 *μ*L) having an inoculum size of approximately 1 × 10^6^ CFU/mL. The final concentrations per well were 500, 250, 125, 64, and 32 *μ*g/mL. The microplates were sealed and incubated at 37°C for 24 h. After incubation, 50 *μ*L of a 2.5% solution of the biological indicator TTC (Triphenyl Tetrazolium Chloride) solution was added, and the plates were incubated again for 2 h to visualize growth inhibition. The lowest concentration of the sample that inhibited the bacterial growth (colourless) after incubation was taken as the MIC. Vancomycin and DMSO were used as positive and negative controls, respectively.

### 2.5. Cytotoxic Assay

#### 2.5.1. Mammalian Vero Cell Line Culture Condition

Vero cell line (ATCC CCL-81) was cultured at 37°C, 5% CO_2_ in DMEM medium (GIBCO) supplemented with 10% heat-inactivated fetal bovine serum (GIBCO) and 0.1 mg/mL streptomycin (GIBCO), and 100 U/mL penicillin (GIBCO).

#### 2.5.2. Cell Viability by LDH Assay

To evaluate the toxicity of extracts, Vero cell line was incubated with samples (ethanolic crude extracts from leaves and stems, hexanic, ethyl acetate, and butanol extracts from fruits) for 24 hours and cell viability measured using LDH assay (Doles). In brief, 5 × 10^4^ cells/well were seeded in a 96-well microplate and incubated for 24 hours to attach. In the following day, cells were washed with PBS, and fresh media DMEM without serum were replaced containing the plant extract at different concentrations (500–31.25 *μ*g/mL). Plates were incubated for further 24 hours and LDH activity measured by colorimetric assay using spectrophotometer (micronal-B582) at 510 nm. As control for maximum LDH release, cells were treated with 0.1% triton-X100 in DMEM medium for 10 min before running the assay. To determine the normal LDH release, cells were cultured in serum-free medium in presence of DMSO. Cell viability was determined using absorbance of treated cells at DMSO as a reference for 100% viability (absorbance of extract-treated cells × 100/absorbance of DMSO-treated cells). 

### 2.6. Statistical Analysis

For antibacterial assay, statistical analysis was performed by ANOVA (one-way Anova) with 95% confidence interval, using the GraphPad Prism 5.0 software package. Differences were considered significant when *P* values were ≤0.05. Vero cell viability (%) was determined by averaging three repeated experiments and IC_50_, representing the concentration at which cell viability was reduced by 50%, was calculated by linear regression using the GraphPad Prism 5.0 software package.

## 3. Results and Discussion

Analysis of the chromatogram obtained from the hexanic extract from fruits of *M. subsericea* indicated the elution of 20 compounds. Substances with retention time (min) of 16.84, 16.99, 18.71, and 18.94 corresponded, respectively, to hexadecanoic acid (A) (5.41%), hexadecanoic acid ethyl ester (B) (3.57%), (E)-9-octadecenoic acid ethyl ester (C) (3.95%), and octadecanoic acid ethyl ester (D) (1.45%). These compounds were identified by comparison of their MS fragmentation pattern with NIST mass spectra. 

**Figure 2:**
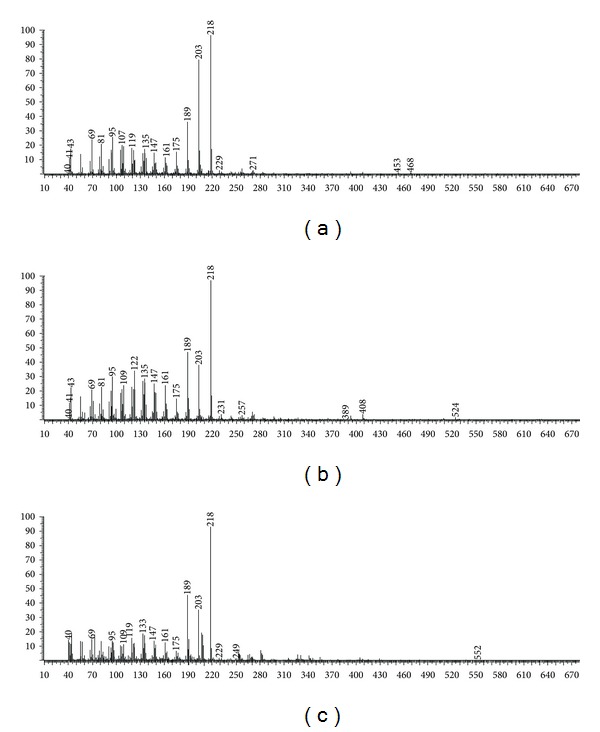
Fragmentation pattern for Δ12-oleane/Δ12-ursane series. (a) Beta-amyrin acetate. (b) Alpha-amyrin caproate. (c) Alpha-amyrin caprylate.

On another study, we described the obtainment and identification of a mixture containing beta-amyrin acetate and alpha-amyrin acetate from edible fruits of this species [13]. Thus, comparison of the previously fragmentation pattern obtained for these substances confirmed the major substances, with retention time (min) of 34.63 and 36.15, as beta-amyrin acetate (E) (10.27%) and alpha-amyrin acetate (F) (42.34%), respectively. The substances with retention time (min) of 53.31/56.55 and 71.70/76.99 also showed a typical fragmentation pattern for pairs of triterpenes from the Δ12-oleane/Δ12-ursane series. The characteristic peaks at *m/z *218 (base peak), 203 and 189 due to Retro-Dials-Alder fragmentation [14] were observed for these substances. Beta-amyrin type triterpenes presented peak at *m/z* 203 higher than peak at *m/z *189, while alpha-amyrin type triterpenes showed an equal abundance (Figure 2). According to Oyo-Ita et al. [15], the amyrin caproates have molecular ion peak (M^+^) at *m/z *524, followed by loss of CH_3_ or the acid moiety to *m/z *509 and 408, respectively. Thus, the substances with retention time (min) of 53.31 and 56.55 could be identified as beta-amyrin caproate (G) (5.46%) and alpha-amyrin caproate (H) (7.26%). The mass fragment at *m/z* 408, due to the loss of 144 (caprylic acid) mass unit from the molecular ion peak (M^+^) at *m/z *552 was in accordance with literature data [16, 17] and suggested substances with retention time (min) of 71.70 and 76.99 as beta-amyrin caprylate (I) (2.44%) and alpha-amyrin caprylate (J) (5.04%), respectively.

**Figure 3:**
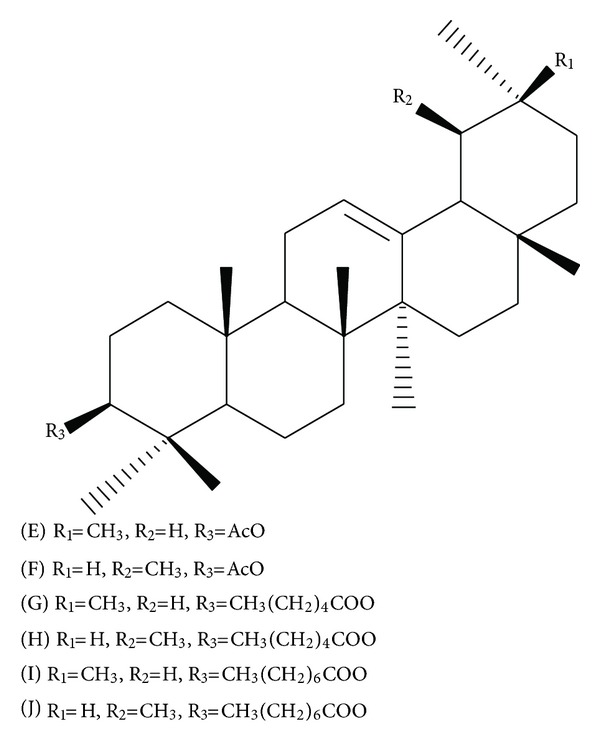
Chemical structures of the amyrin esters: beta-amyrin acetate (E), alpha-amyrin acetate (F), beta-amyrin caproate (G), alpha-amyrin caproate (H), beta-amyrin caprylate (I), and alpha-amyrin caprylate (J) from the hexanic extract from fruits of *Manilkara subsericea. *

It is interesting for the chemotaxonomic consideration that several studies carried out for *Manilkara *species, such as *Mimusops littoralis* Kurz (*Manilkara littoralis* (Kurz) Dubard) [18], *Mimusops manilkara* G.Don (*Manilkara kauki* (L.) Dubard) (Misra and Mitra, 1969), and *Mimusops hexandra* Roxb (*Manilkara hexandra* (Roxb.) Dubard) [19] indicated the presence of triterpene esters. Thus, the chemical substances identified on the hexanic extract from fruits of *M. subsericea *(Figure 3) are in accordance with the chemical markers of the genus *Manilkara*.

**Figure 4:**
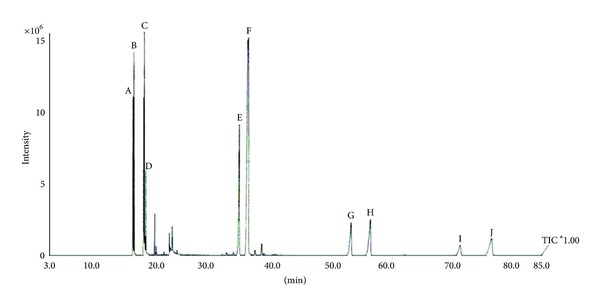
CG-FID chromatogram of the hexanic extract from fruits of *Manilkara subsericea*, Sapotaceae. A: hexadecanoic acid (5.41%), B: hexadecanoic acid ethyl ester (3.57%), C: (E)-9-octadecenoic acid ethyl ester (3.95%), D: octadecanoic acid ethyl ester (1.45%), E: beta-amyrin acetate (10.27%), F: alpha-amyrin acetate (42.34%), G: beta-amyrin caproate (5.46%), H: alpha-amyrin caproate (7.26%), I: beta-amyrin caprylate (2.44%), J: alpha-amyrin caprylate (5.04%). For analysis conditions see Materials and Methods, Section 2\tmspace +\thinmuskip {.1667em}2.3.

The identified substances corresponded to 87.19% of the total relative composition of the hexanic extract from fruits of *M. *subsericea. The individual amounts of each substance are illustrated in Figure 4. Furthermore, to our knowledge, this is the first time that the beta- and alpha-amyrin caproates and caprylates are described for the *Manilkara subsericea *species. 

Antibacterial assay was performed against *Staphylococcus aureus *ATCC25923 and *Escherichia coli *ATCC36298. There were significant differences (*P* < 0.05) in the antibacterial activity of ethanolic crude extract from leaves (7 ± 0.28 mm), ethanolic crude extract from stems (8 ± 0 mm), and hexanic extract from fruits (6 ± 0 mm), which were considered active against *S. aureus*. (Table 1). Ethyl acetate and butanol extracts from fruits did not inhibit the *S. aureus *growth. All extracts were considered inactive against *E. coli*. (Table 1).

The extracts that exhibited antimicrobial activity during the disk diffusion method were evaluated for their *Minimum inhibitory concentration* (MIC). All extracts tested inhibited the bacterial growth of the *S. aureus* strain with MIC of 250 *μ*g/mL. Terpenoids are active against bacteria but the mechanism of action of terpenes is not fully understood, although it is speculated to involve membrane disruption by lipophilic compounds [20]. The isomeric mixture of beta-amyrin and alpha-amyrin is known by its antimicrobial activity [21], and 72.81% of the relative amount of the hexanic extract from fruits is constituted by esters of these substances. Beta- and alpha- amyrin acetates are also known by their anti-inflammatory activity and also inhibitory effects on Epstein-Barr virus early antigen (EBV-EA) in Raji cells [22]. Furthermore, according to Hichri et al. [23], the triterpene beta-amyrin acetate was able to inhibit the bacterial growth of the *Staphylococcus aureus *ATCC25923 reference strain at 90 *μ*g/mL. Thus, our results suggest that the antibacterial activity found in the hexanic extract from fruits may be modulated by the beta- and alpha- amyrin esters identified.

**Figure 5:**
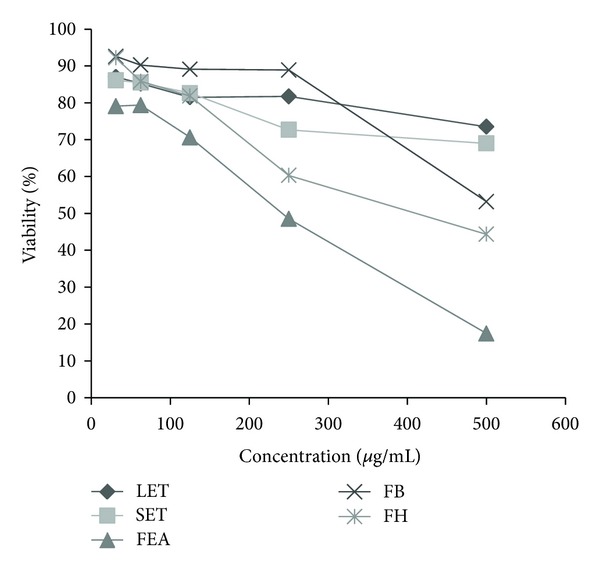
Vero cell viability in the presence of extracts for 24 h measured by LDH assay. LET: ethanolic crude extract from leaves, SET: ethanolic crude extract from stems, FEA: ethyl acetate extract from fruits, FB: butanol extract from fruits, FH: hexanic extract from fruits.

All tested extracts demonstrated weak cytotoxic effects on the mammalian Vero cells. The Cell viability on treatment with hexanic and ethyl acetate extracts from fruits was 69.66% and 56.07% in concentration of 250 *μ*g/mL, respectively (Figure 5). Ethanolic crude extract from leaves (1658 *μ*g/mL; 1164–2525) had highest IC_50_ value, followed by ethanolic crude extract from stems (1112 *μ*g/mL; 757–2525), butanol extract from fruits (683.4 *μ*g/mL; 451–2200), hexanic extract from fruits (482.6 *μ*g/mL; 385–677), and ethyl acetate extract from fruits (307.6 *μ*g/mL; 276–346). 

The triterpene beta-amyrin acetate was reported to have cytotoxicity against A2780 ovarian cancer cell line with IC_50_ of 12.1 *μ*g/mL [24]. This compound was not considered active against A549, SK-OV-3, SK-MEL-2, XF498, and HCT15 cancer cell lines [25]. Moreover, some beta- and alpha- amyrin esters were able to induce cell apoptosis in HL-60 leukemia cells [26]. 

## 4. Conclusions

Although *Manilkara subsericea* fruits are used as food, to our knowledge, only one article regarding its phytochemicals and biological activities was published [13]. The present study describes the identification of a high percentage of substances from the hexanic extract from edible fruits of *Manilkara subsericea*, in which beta- and alpha- amyrin caproates and caprylates are reported for the first time for this species. Our results suggest that this hexane extract from fruits and ethanolic crude extract from leaves and stems presented antimicrobial activity against *S. aureus* ATCC25923. In addition, these extracts had low cytotoxicity on Vero cells, in the same concentration which inhibited *S. aureus* growth. Several biological studies are carried out for mixtures of beta- and alpha- amyrin type triterpenes [21, 27, 28], since their separation by conventional chromatographic methods is quite difficult [29]. 

## Figures and Tables

**Table 1:** Means of the inhibition halos (mm ± SD) for *Staphylococcus aureus *and *Escherichia coli* tested with extracts (100 mg/mL) from *Manilkara subsericea*. LET: ethanolic crude extract from leaves, SET: ethanolic crude extract from stems, FH: hexanic extract from fruits, FEA: ethyl acetate extract from fruits, FB: butanol extract from fruits. Vanc: vancomycin (30 *μ*g), ampicillin: (30 *μ*g).

	Inhibition halo (mm) ± SD	
	*Staphylococcus aureus *	*Escherichia coli *
LET	7 ± 0.28^b^	0^b^
SET	8 ± 0^c^	0^b^
FH	6 ± 0^d^	0^b^
FEA	0^e^	0^b^
FB	0^e^	0^b^
Vanc	18 ± 0.28^a^	Not tested
Amp	Not tested	32 ± 7^a^

Means in the same column with different superscripts are significantly different (*P* < 0.05).
